# Carbohydrate-Restriction with High-Intensity Interval Training: An Optimal Combination for Treating Metabolic Diseases?

**DOI:** 10.3389/fnut.2017.00049

**Published:** 2017-10-12

**Authors:** Monique E. Francois, Jenna B. Gillen, Jonathan P. Little

**Affiliations:** ^1^University of British Columbia Okanagan, Kelowna, BC, Canada; ^2^University of Toronto, Toronto, ON, Canada

**Keywords:** high-intensity interval training, glycemic control, metabolism, cardiometabolic disease, exercise, low-carb diets

## Abstract

Lifestyle interventions incorporating both diet and exercise strategies remain cornerstone therapies for treating metabolic disease. Carbohydrate-restriction and high-intensity interval training (HIIT) have independently been shown to improve cardiovascular and metabolic health. Carbohydrate-restriction reduces postprandial hyperglycemia, thereby limiting potential deleterious metabolic and cardiovascular consequences of excessive glucose excursions. Additionally, carbohydrate-restriction has been shown to improve body composition and blood lipids. The benefits of exercise for improving insulin sensitivity are well known. In this regard, HIIT has been shown to rapidly improve glucose control, endothelial function, and cardiorespiratory fitness. Here, we report the available evidence for each strategy and speculate that the combination of carbohydrate-restriction and HIIT will synergistically maximize the benefits of both approaches. We hypothesize that this lifestyle strategy represents an optimal intervention to treat metabolic disease; however, further research is warranted in order to harness the potential benefits of carbohydrate-restriction and HIIT for improving cardiometabolic health.

## Introduction

Type 2 diabetes (T2D) is one of the fastest growing, yet largely preventable chronic diseases worldwide (1). In addition to the hallmark feature of impaired glucose control, progression of T2D presents with a number of co-morbidities, increasing risk of premature mortality. Of particular concern is the high prevalence of atherosclerotic cardiovascular disease (CVD), which accounts for ~80% of T2D-related co-morbidities (2, 3). Furthermore, a clustering of cardiovascular risk factors tend to manifest well before the diagnosis of T2D, accelerating the progression of CVD (4). These risk factors include central obesity, hypertension, hyperglycemia, and dyslipidemia, collectively coined the metabolic syndrome (5). In addition, physical inactivity and/or low-cardiorespiratory fitness are emerging as important modifiable risk factors for CVD and T2D (6). Importantly, both exercise and dietary strategies can help prevent, or slow the progression of, T2D-related co-morbidities. In this regard, lifestyle interventions incorporating both diet and exercise prescriptions remain at the frontline of therapeutic options for treating metabolic disease (7–9).

The main treatment goal of metabolic diseases, including T2D and the metabolic syndrome, is the prevention of atherosclerotic CVD (10, 11). To date, management of T2D and associated co-morbidities depends upon pharmacological interventions and the implementation of lifestyle changes (9, 11). Generally, public health guidelines encourage weight loss by recommending a calorie-restricted diet that is low in fat and added sugar, as well as performing at least 150 min of moderate-intensity physical activity each week (10, 11). While this general approach may be effective for the prevention of T2D (12), it does not appear to prevent, or alleviate, the burden of CVD in those with overt T2D [(8), LOOK Ahead trial]. Given the significant burden of CVD and diabetes-related co-morbidities, novel intervention strategies that can reduce cardiovascular risk factors, while imposing minimal side effects, are urgently sought after.

In the growing field of health and exercise science, a number of lifestyle approaches are currently gaining momentum. For example, dietary modifications such as carbohydrate-restriction and time-restricted feeding (including intermittent fasting) are emerging as alternative treatment strategies for persons with, and at risk for, metabolic diseases. Likewise, novel exercise prescriptions including high-intensity interval training (HIIT), and the concept of breaking up sedentary time with light physical activity, have revealed significant promise for improving or mitigating the deleterious effects of an inactive lifestyle. Innovative and effective strategies such as these have reaffirmed the strong influence of lifestyle modification on metabolic disease progression. While some of these individual approaches are beginning to inform public health guidelines (9), it is our belief that combining both diet and exercise modification will synergistically aid in the management of metabolic disease. In this regard, carbohydrate-restriction is loosely defined as restricting carbohydrates to less than 30% of caloric intake, and HIIT is characterized by brief periods of high-intensity exercise interspersed with low-intensity exercise for recovery. We acknowledge there are several other promising lifestyle interventions that we have not mentioned, however, the focus of this paper is to highlight the propensity for synergistic effects when carbohydrate-restriction and HIIT are combined. As with any lifestyle intervention, adherence is critical, and behavior change research on how to implement and support both HIIT and carbohydrate-restriction is needed. Initial work on adherence to HIIT is showing promise (13) and low-CHO diets appear to have similar adherence to other diets (14), but it will be necessary to assess the tolerability and adherence to our hypothesized approach moving forward.

## Hypothesis

In light of recent evidence, we hypothesize that the novel combination of a carbohydrate-restricted diet and HIIT represents a promising lifestyle strategy for the treatment of T2D. The purpose of this *Hypothesis & Theory* piece is to briefly summarize the evidence that supports the individual therapeutic benefits of carbohydrate-restriction and HIIT, as well as present the idea that combining these approaches may represent the most potent lifestyle therapy for this costly disease.

## Carbohydrate-Restriction in T2D

Due to the growing prevalence of metabolic disease and the apparent ineffectiveness of the current dietary guidelines (15, 16), alternative dietary approaches need to be considered. There is increasing evidence from scientific research [reviewed in Ref. (17)] and patient groups (UK Diabetes low-carb initiative; www.diabetes.org.uk) to support carbohydrate-restriction as a primary dietary treatment strategy for individuals with T2D. It has long been known that a diet high in carbohydrate elevates postprandial hyperglycemia and insulin responses, together accelerating the progression of T2D and atherosclerotic CVD (18–20). To this end, diets low in carbohydrate was recommended for T2D in 1800s and during the early twentieth century. More recently, low-carbohydrate diets are recognized in the ADA medical nutrition therapy guidelines (21) although the official dietary guidelines for individuals with T2D still do not advocate a low-carbohydrate approach. In comparison with standard low-fat caloric restrictive dietary interventions, energy-matched diets that restrict carbohydrates to <30 g/day have been shown to result in greater reductions in HbA_1c_ and fat mass (22, 23), as well as superior improvements in the blood lipid profile (24, 25). Typically, low-carbohydrate diets are not explicitly prescribed to be hypocaloric, but due to the satiating effects of protein and fat, energy intake is often lower (22, 26–28). However, while the energy intake is reduced relative to participant’s habitual intake or when a low-fat diet is prescribed, low-carbohydrate diets reduce energy intake relative to energy requirements (26–28). For example, Boden et al. (26) observed a 1,027 cal/day reduction in energy intake when patients with T2D followed an *ad libitum* low-carbohydrate diet for 14 days; interestingly, the energy intake was reduced to a level that was appropriate to their weight. Moreover, the benefits of low-carbohydrate diets can occur without weight loss (29, 30). For a comprehensive review of carbohydrate-restriction for the management of T2D, an interested reader is directed to Feinman et al. (28).

While optimal guidelines for carbohydrate-restriction are not established, a carbohydrate-restrictive diet generally constitutes <30% of caloric intake from carbohydrate food-sources (approximating <130 g/day) (31). Very low-carbohydrate diets on the other hand, often referred to as ketogenic diets, involve more extreme reductions in carbohydrate of less than ~30 g/day to permit nutritional ketosis (32). The optimal amount of carbohydrate in the diet (degree of carbohydrate-restriction) likely depends on the state of insulin resistance of the individual. For example, Cornier et al. (33) reported greater weight loss in insulin-sensitive individuals following a high- compared with a low-carbohydrate hypocaloric diet, whereas the opposite was true for insulin-resistant individuals (i.e., greater weight loss and improved insulin sensitivity on a low-carbohydrate hypocaloric diet). The implications of this study are noteworthy, given that more than 35% of adults are insulin resistant (34). Therefore, speculatively, more insulin-resistant individuals, particularly those with T2D, may require greater degrees of carbohydrate-restriction for diet-induced improvements in metabolic health.

In longer-term interventions, a carbohydrate-restricted diet appears highly effective at promoting weight loss in patients with T2D (25, 27, 35). This dietary strategy has also been shown to reduce visceral adiposity (22, 27, 36), and lower medication requirements in adults with T2D (25, 37). Such reductions in central adiposity, insulin resistance, and hyperglycemia are central to preventing the development of CVD (38). Importantly, even in the absence of weight loss, carbohydrate-restrictive diets have also been shown to improve glycemic control (29, 39). For example, Gannon and colleagues found that despite no change in total body mass, an isocaloric diet comprising 30% protein, 50% fat, and 20% carbohydrate reduced HbA_1c_ by 2% (absolute reduction) and improved fasting and postprandial blood glucose control in patients with T2D (30, 40). Such improvements in glucose control in the absence of weight loss may be due to improved insulin sensitivity and/or beta-cell function. Indeed, Boden et al. (26) showed that just 2 weeks of an isocaloric low-carbohydrate diet improves insulin sensitivity by 75% in T2D individuals, using the gold standard hyperinsulinemic euglycemic clamp technique. Although we are unaware of any direct evidence for improved beta-cell function with carbohydrate-restriction in individuals with T2D, associative evidence supports that providing beta-cell rest by removing hyperglycemia can reverse the insulin secretory defects present in animal models of T2D (41, 42).

## Exercise in T2D: It is a HIIT!

Physical inactivity presents one of the greatest public health concerns of our time (43). Indeed inactivity, or perhaps more accurately termed insufficient physical activity, is the fourth leading cause of death (44). Physical activity is broadly defined as any bodily movement produced by skeletal muscle, encompassing both activities of daily living and structured exercise. Exercise is defined as purposeful physical activity carried out to sustain or improve health or fitness, and if provided in a sufficient stimulus (dependent upon intensity and duration) is very effective to improve cardiometabolic health (45). The protective and therapeutic effects of regular exercise for the prevention of many chronic diseases are well-established and have been comprehensively reviewed by Pedersen and Saltin (46).

In order to achieve such health benefits, it is recommended that 150 min of moderate-to-vigorous intensity physical activity be performed each week. Perhaps one of the greatest health benefits of a regular exercise regimen is an improvement in cardiorespiratory fitness (47). Indeed, improving cardiorespiratory fitness is associated with a lower risk of both all-cause and CVD mortality (48, 49). The positive relationship between regular exercise and improvements in cardiorespiratory fitness appears to be intensity-dependent, however, with higher intensity exercise conferring larger improvements in fitness (50, 51). In fact, several systematic reviews and meta-analyses have reported that in comparison with moderate-intensity exercise, exercise performed at a higher intensity elicits greater improvements in markers of cardiovascular, and metabolic health in individuals with T2D (52–55). In agreement, a recent review by Baldi et al. (56), suggested that even public health guidelines, which recommend moderate-intensity continuous training (MICT), may not provide a sufficient stimulus for improving cardiovascular function. Thus, performing exercise at a vigorous intensity (relative to the individuals baseline fitness level) may be required to improve cardiovascular and metabolic health (57).

High-intensity interval training, which involves alternating periods of relatively intense exercise with periods of rest or low-intensity exercise for recovery (58, 59), is an attractive strategy enabling vigorous-intensity exercise to be incorporated in an exercise program. In comparison with MICT, HIIT is performed in an intermittent pattern, which results in only brief periods of relatively high-intensity effort, followed by recovery periods. Importantly, HIIT is a highly potent strategy to improve cardiorespiratory fitness (59). Indeed, a recent meta-analysis reported that HIIT-induced improvements in cardiorespiratory fitness were nearly one metabolic equivalent (+3 ml/kg/min) higher than in response to MICT in individuals with lifestyle induced chronic disease (59). Improvements of this magnitude correspond to a ~15% greater risk reduction for CVD morality (49), highlighting the potency of HIIT for reducing cardiovascular risk.

Various iterations of HIIT have been tested in small trials of T2D patients with reported benefits to cardiorespiratory fitness, glucose control, hepatic fat, vascular function, and body composition. While direct comparisons with standard care MICT are less common, recent investigations have reported superior improvements in many of these risk factors following HIIT (59–61). For example, an HIIT protocol involving 4× 4 min intervals at ~90% of maximal aerobic capacity, interspersed with 3 min of recovery has consistently yielded superior cardiovascular adaptations compared with an energy-matched MICT protocol. Following 12 weeks of training, endothelial function (62, 63), diastolic and systolic function (63) as well as cardiorespiratory fitness (62–64) were improved to a greater extent following HIIT compared with MICT.

Given the prevailing hyperglycemia among patients with T2D, the impact of HIIT on glycemic control has received much attention (63–66). In a landmark study, Karstoft et al. (65, 67) compared 60 min of interval-walking 5 days per week, to an energy-expenditure matched continuous-walking protocol in T2D. The interval-walking group performed 3 min periods of fast walking (above 70% VO_2_peak), followed by 3 min of slow walking (below 70% VO_2_peak) for 60 min three times per week, while the continuous protocol involved 60 min of walking at 55% VO_2_peak. Following 16 weeks of training, the interval-walking group displayed greater reductions in mean 24-h blood glucose concentration assessed with continuous glucose monitoring, which were accompanied by superior improvements in cardiorespiratory fitness and body composition. In a follow-up publication in the same participants, authors reported improved insulin sensitivity in the interval-walking group only, as measured by hyperglycemic clamp with glucose tracers. These superior improvements in glucose control are reinforced by a recent meta-analysis demonstrating that HIIT improves fasting blood glucose and HbA_Ic_ to a greater extent than MICT in individuals at risk for, or afflicted with, T2D (60). Specifically, in those with T2D, HIIT was found to lower fasting glucose and HbA_1c_ by 0.92 mmol/l and 0.5%, respectively, which is of comparable magnitude to pharmacological-induced improvements in glycemia (68). The pronounced improvements in glucose control following HIIT are likely mediated by many factors, some of which may include greater beta-cell function (67, 69), improved skeletal muscle insulin signaling (62, 67), and reductions in total body (62, 64, 65) and hepatic (66, 70) fat content.

While these findings are compelling, the time commitment associated with many HIIT protocols is often higher (150–300 min/week) than that obtained by the general population, which questions whether such exercise is attainable for many individuals who report a “lack of time” as a barrier to regular exercise (71). With this in mind, low-volume HIIT protocols involving a reduced exercise volume and time commitment may represent a viable strategy. We have previously shown that 2 weeks of HIIT involving six sessions of 10× 60 s cycling intervals at ~90% of maximal heart rate, interspersed with 60 s of rest, improved 24-h mean blood glucose concentration, and lowered postprandial glucose excursions in patients with T2D (72). Using this same protocol, Madsen et al. (69) reported a 0.5% reduction in HbA_1c_ and increased glucose tolerance and beta-cell function after 8 weeks of training in T2D. In addition to improvements in glycemic control, we (73) and others (74) have reported increases in cardiorespiratory fitness and total body lean mass as well as reductions in body fat following 12 weeks of low-volume HIIT in patients with T2D. These findings are quite intriguing, given that total exercise time was only ~30 min/week within a 75-min weekly time commitment. Importantly, low-volume HIIT has been reported to be enjoyable (13, 75, 76), which may be attributable to the low-time commitment and/or short interval duration (77).

The mechanisms by which HIIT improves metabolic and cardiovascular health are likely multifaceted, and may relate to the high rates of muscle fiber recruitment, rapid muscle glycogen depletion, and repetitive shear stress during exercise (78, 79). The intermittent cardiorespiratory and metabolic demands imposed also appear to be important in regulating chronic adaptations to HIIT (79). Indeed, it has been suggested that training with alternating intensity, and not solely measuring training volume and mean intensity, is important for improving cardiovascular and metabolic health in individuals with T2D (65, 67, 80).

## Combining Carbohydrate-Restriction and HIIT for Improved Cardiometabolic Health

**Figure 1:**
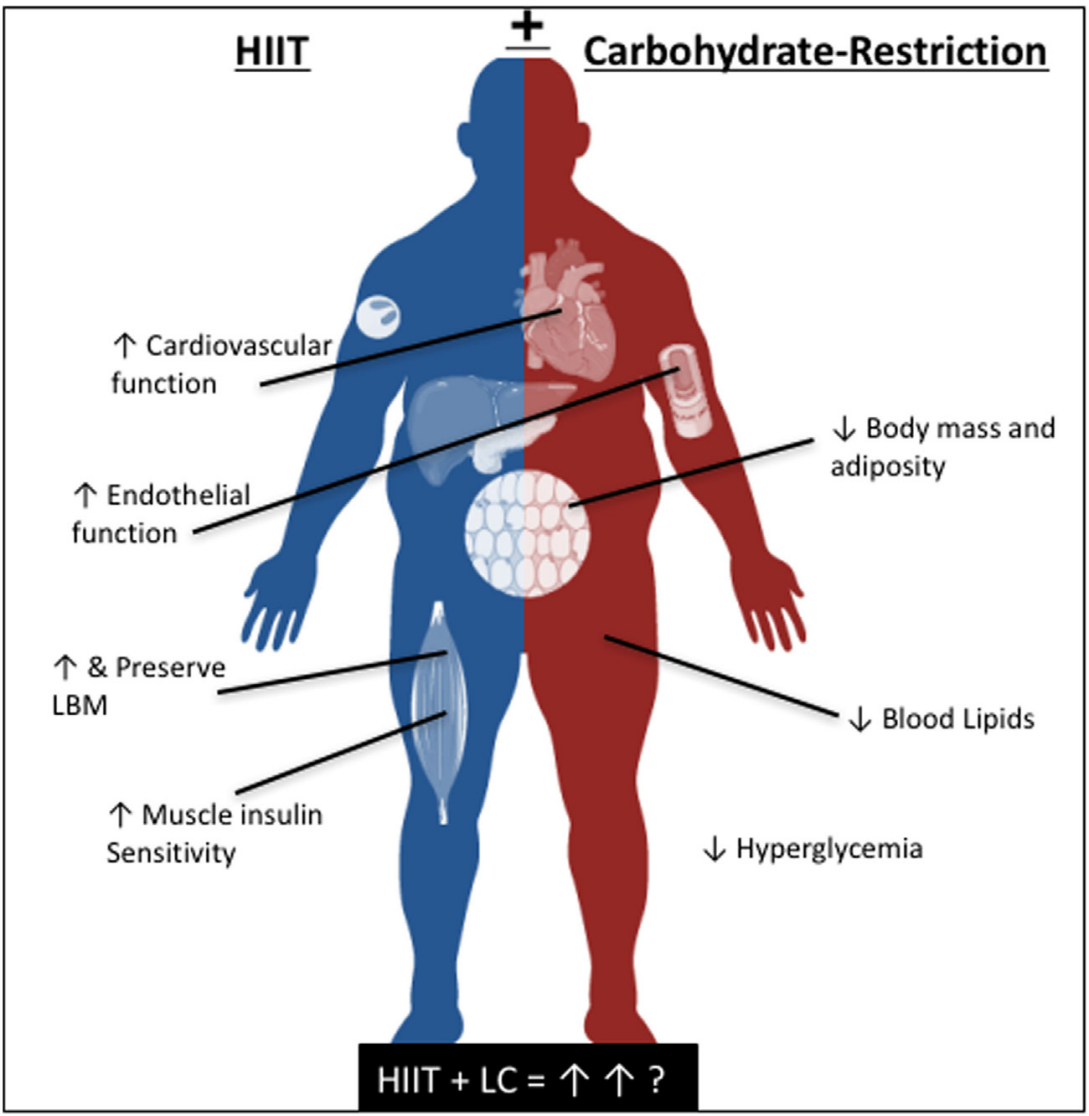
The independent, and proposed reciprocal benefits of high-intensity interval training and carbohydrate-restriction for cardiometabolic health.

While the independent improvements in cardiometabolic health following carbohydrate-restriction and HIIT have been well investigated, the combined impact of these lifestyle strategies in patients with T2D has yet to be explored. It is our hypothesis that supplementing a carbohydrate-restrictive diet with HIIT, or likewise, strategically limiting carbohydrate availability during HIIT, may enhance the therapeutic effects of either intervention alone (Figure 1). Specifically, we believe that a combined approach will synergistically improve the acute and chronic regulation of glycemic control and endothelial function while maximally improving cardiorespiratory fitness, resulting in the optimal lifestyle strategy to limit the progression of T2D, and related co-morbidities.

### Adding HIIT to a Carbohydrate-Restrictive Diet

While a carbohydrate-restrictive diet effectively reduces hyperglycemic excursions and improves glycemic control in patients with T2D (29, 39), a perceived limitation of this dietary approach is that it is inevitably higher in dietary fat. Studies in rodents (81, 82) and epidemiology evidence in humans suggests that a high-fat diet (which is also low in carbohydrate) promotes insulin resistance (83, 84). Specifically, it appears that in the short-term, high-fat diets (for ≤1 week) in humans may impair glucose tolerance (when tested by providing a high-glucose load), at least from trials in healthy adults (85, 86). In healthy insulin-sensitive participants, a reduction in insulin sensitivity and/or glucose tolerance following a switch to a low-carbohydrate high-fat diet is likely an adaptive response as the body transitions over to higher baseline fat utilization (87, 88). However, individuals with obesity and insulin resistance experience some degree of metabolic inflexibility; an inability to modulate daily fat and carbohydrate oxidation based upon substrate availability (89). Consequently, exaggerated blood glucose and lipid responses to meals are prevalent in individuals with insulin resistance (90). This suggests that if one is to follow a low-carbohydrate diet, it must be followed consistently (i.e., no “cheating” or consumption of high-glycemic index foods) or that exercise should be incorporated to mitigate any detrimental effects on insulin sensitivity. Indeed, the most important determinant of the effectiveness of dietary interventions is adherence (91). However, we have shown that a single session of low-volume HIIT performed after breakfast lowers postprandial glucose excursions throughout the day in patients with T2D (92) and “small snacks” of interval exercise distributed before meals significantly lowers postprandial hyperglycemia and mean glucose concentration over 24 h (93). Therefore, exercise, of a sufficient stimulus, strategically timed to dispose of postprandial glucose and/or to increase fat utilization is a highly effective strategy to reduce postprandial excursions. In considering the glycemic response to a meal is primarily determined by the quantity and type of carbohydrate (94), restricting carbohydrate inevitably will lower postprandial glucose and insulin excursions (95, 96). Thus, it would appear that the combination of carbohydrate-restriction with HIIT may promote synergistic improvements for acutely reducing postprandial glucose spikes, increasing muscle glucose uptake, and augmenting insulin sensitivity.

Furthermore, exercising before or after a high-fat meal has been shown to ameliorate the detrimental effects on endothelial function (97–99). Endothelial function is an important prognostic indicator of cardiovascular health as the endothelium is the barrier protecting the artery from thrombosis, inflammation, and stiffening (100, 101). Postprandial increases in lipids and glucose are independent risk factors for CVD (102). The postprandial impairment in endothelial function may be related to excessive postprandial hypertriglyceridemia, inflammation, and oxidative stress following the meal (103, 104). Given that several studies have supported the notion that “high-fat” meals [which are often also high in refined carbohydrate (105–107)] can promote endothelial dysfunction, there appears to be some apprehension around adopting a low-carbohydrate high-fat diet. However, this is despite the fact that high-carbohydrate meals can elicit similar dysmetabolism and endothelial dysfunction (105). Regardless, if a typical low-carbohydrate high-fat meal does acutely impair endothelial function this may not be an ideal strategy for participants at elevated CVD risk. However, Tyldum et al. (99) reported that a single bout of HIIT, but not MICT, performed ~16-h prior to a high-fat meal protected against endothelial dysfunction. Interestingly, this was tightly related to exercise-induced increases in antioxidant capacity with HIIT, as measured by the colorimetric total antioxidant capacity assay (99). Tjønna et al. (108) also showed that endothelial function and nitric oxide bioavailability were increased ~72 h after an acute session of HIIT in individuals with T2D. Collectively, it appears that exercise can negate the detrimental effects of a high-fat meal on endothelial function, with HIIT as a promising strategy to improve endothelial function. Therefore, strategically timing HIIT sessions could reduce any potential negative effects of low-carbohydrate high-fat meals that are often incorporated into carbohydrate-restricted meal plans.

Lastly, over the long term, reductions in fat mass and increases in lean mass appear critical for sustained improvements in metabolic health following lifestyle interventions (109, 110). In this regard, the combination of diet and exercise may be superior for preserving lean mass and reducing body fat in patients with T2D (111, 112). Energy restriction interventions, in the absence of exercise, often result in the loss of lean body mass in addition to fat loss (113, 114). Given the strong relationship between lean body mass, metabolic health, and functional capacity (115), preserving lean body mass with exercise during any energy-restricted diet (whether low-carbohydrate or not) is of primary importance. Moreover, preserving lean body mass in individuals with T2D is particularly important given the accelerated loss of skeletal muscle with insulin resistance, and the concomitant worsening of glycemic control with decreased skeletal muscle mass (116, 117). In view of this, HIIT has recently been shown to increase skeletal muscle protein synthesis of young and older adults, an effect linked to improved insulin sensitivity, and mitochondrial function (118). Relative to MICT and resistance training, HIIT resulted in the greatest increase in gene expression for mitochondrial function, muscle growth, and insulin signaling pathways in older adults (118). In both obese adults and those with and T2D, HIIT has been shown to reduce fat mass and increase lean mass (62, 64, 65, 69, 73, 74). Although the impact of a low-carbohydrate diet combined with HIIT has not been adequately studied, there is evidence that a low-carbohydrate diet promotes favorable changes in body composition when combined with resistance training in women with obesity (119). Thus, the combination of HIIT with a low-carbohydrate diet may help to preserve or increase lean mass in individuals with T2D.

## Exercise and Nutrient Interactions: Adding Carbohydrate-Restriction to HIIT

It is well known that nutrient ingestion in-and-around the exercise period impacts the molecular signaling and thus metabolic responses to exercise (120). Accumulating evidence has revealed that nutrient availability, in addition to exercise components (e.g., mode, intensity, and duration), play a putative role in determining the adaptive response to exercise training (121). Indeed energy status (whether surplus or deficit) has been shown to modify the neuroendocrine, acute molecular signaling, and gene transcription response to exercise (121, 122). For example, low-skeletal muscle glycogen prior to exercise augments AMPK signaling and up-regulates the transcription of PGC1a and mitochondrial enzymes (123–125). It has also been suggested that delaying glycogen restoration after exercise may enhance the adaptive response for proteins involved in glucose uptake (i.e., glucose transporter GLUT-4) (126, 127). Therefore, restricting carbohydrates and/or training fasted may increase the adaptive (i.e., training-induced increase in metabolic signaling) response to exercise (121, 128, 129). In contrast, providing carbohydrate in proximity to exercise can in fact blunt the activation of key regulatory genes for metabolizing fat postexercise (123). Thus, it is tempting to speculate that combining HIIT with a low-carbohydrate diet in T2D could enhance molecular signaling and metabolic adaptations in skeletal muscle.

In support of this, Newsom et al. (130) reported that an energy deficit after exercise does not contribute to the exercised-induced improvements in insulin sensitivity if ample carbohydrate is provided. However, withholding carbohydrate (but providing ample energy) postexercise resulted in improved insulin sensitivity the following day. Furthermore, restricting carbohydrate before and between interval training sessions has been shown to augment the cellular signaling and upregulate skeletal muscle metabolic enzyme adaptations in young healthy participants (131, 132). This suggests that strategically restricting carbohydrate during HIIT could potentiate exercise-induced skeletal muscle adaptations in patients with T2D. To our knowledge, however, this strategy has not been examined in clinical populations. In the only study we are aware of, Sartor et al. (133) reported increased resting fat oxidation and cardiorespiratory fitness following a 2-week HIIT plus carbohydrate-restricted diet intervention in obese men. However, changes in insulin sensitivity and glucose control were not reported and thus future long-term studies are warranted.

## Perspectives for Future Research

Carbohydrate-restriction and HIIT have independently been shown to improve several indices of cardiometabolic health. Taken together, it is our opinion that an adjunct therapy incorporating both carbohydrate-restriction and HIIT would be a particularly effective treatment for metabolic and CVDs (Figure 1). However promising, several key questions require further research, namely (i) the level of carbohydrate-restriction that is effective, safe, and feasible for different individuals over the long-term and (ii) the most effective HIIT protocol to complement a carbohydrate-restrictive diet. For example, low-volume HIIT protocols have been shown to improve a host of cardiometabolic risk factors, and are well-tolerated and enjoyed by individuals with T2D (13, 70, 73), however, future research on the integration of HIIT with carbohydrate-restriction is needed. In this regard, using rating of perceived exertion as an indication of exercise intensity may be more appropriate than heart rate (70) given the tolerance to HIIT during carbohydrate-restriction may be reduced. Sartor et al. (133) showed promising effects of carbohydrate-restriction with interval training (4× 4-min intervals) over 14 days in obese individuals. However, further research is needed in this area to determine the exercise dose that is feasible and well-tolerated during carbohydrate-restriction. In this regard and with the advancement of personalized and precision medicine, the development of an algorithm that could be used to adjust the level of carbohydrate-restriction and corresponding HIIT protocol according to age, underlying insulin resistance, fitness, preference, and genetics could prove useful. Considering there are many effective HIIT protocols, the “optimal” protocol may in fact be based on preference rather than physiology.

## Conclusion: HIIT with Carbohydrate-Restriction: an Optimal Combination for Individuals with T2D?

We hypothesize that the combination of HIIT with a carbohydrate-restrictive diet may be the most effective strategy to reduce hyperglycemia, improve insulin sensitivity, promote favorable changes in body composition, and preserve (or increase) endothelial function in patients with T2D. While the combination of diet and exercise for the treatment of T2D is self-evident, the optimal combination is yet to be determined. Strategically timing HIIT in proximity to low-carbohydrate high-fat meals may synergistically maximize the benefits of both approaches, as well as minimize any potential negative effects of postprandial lipemia if one is following a low-carbohydrate high-fat diet. Such a strategy is indeed testable and warranted to improve cardiometabolic health and reduce cardiovascular risk in T2D.

## Author Contributions

MF, JG, and JL contributed to the ideas and hypotheses presented in this manuscript; drafted, revised, and edited the manuscript.

## Conflict of Interest Statement

The authors declare that the research was conducted in the absence of any commercial or financial relationships that could be construed as a potential conflict of interest.
